# The impact of intravenous immunoglobulin therapy on resource utilization associated with viral respiratory tract infections

**DOI:** 10.3389/fimmu.2025.1513712

**Published:** 2025-08-06

**Authors:** Antoine Azar, M. Chris Runken, Eric Moughames, Reuben Howden, Joshua Oh, Christopher M. Blanchette

**Affiliations:** ^1^ Division of Allergy and Clinical Immunology, Johns Hopkins University, Baltimore, MD, United States; ^2^ Global Health Economics Outcomes Research & Real-World Evidence, Grifols Shared Services North America, Research Triangle Park, NC, United States; ^3^ Department of Applied Physiology, Health & Clinical Sciences, University of North Carolina at Charlotte, Charlotte, NC, United States; ^4^ Pharmacy Practice and Administration, Rutgers University Ernest Mario School of Pharmacy, Piscataway, NJ, United States; ^5^ Pharmaceutical & Healthcare Marketing, Saint Joseph’s University Erivan Haub School of Business, Philadelphia, PA, United States

**Keywords:** viral respiratory tract infection, immune deficiency, intravenous immune globulin, healthcare resource utilization, immunocompromised patients

## Abstract

**Introduction:**

Immunocompromised patients with moderate to severe viral respiratory tract infections (VRTIs) may benefit from intravenous immune globulin (IVIG) in combination with antivirals. The impact of this therapy on hospital resource utilization is unknown. The purpose of this study was to assess clinical outcomes and hospital resource utilization associated with IVIG use in immunocompromised patients hospitalized with VRTIs.

**Methods:**

Using the Premier Healthcare Database, data from 1,927 inpatients with immune deficiency and acute VRTI were analyzed. Outcomes included measures relevant to hospital resource use and patient death rates. Descriptive statistics were used to measure factors associated with IVIG use across the hospital stay. A logistic regression model adjusted for factors associated with the probability of IVIG use within 48 hours of admission. The propensity score was used to weigh subsequent models to assess the length of stay (total and ICU) using a negative binomial model and logistical regression for inpatient death.

**Results:**

Of the 1,927 patients analyzed, 65 received IVIG. When adjusting for IVIG use within 48 hours of admission and other patient and hospital characteristics, findings showed a significantly shorter hospital length of stay for patients with acute VRTIs when IVIG was given (p = 0.027). The length of ICU stay was also significantly shorter with IVIG use (p = 0.003).

**Discussion:**

Immunocompromised patients with VRTIs who receive IVIG within 48 hours of ICU admission may have a shorter ICU length of stay and shorter overall hospital length of stay thereby possibly decreasing healthcare resource use.

## 1 Introduction

Severe viral respiratory tract infections (VRTIs) are prevalent in hospital intensive care units and are associated with substantial hospital resource use and treatment costs (1–3). These infections are predominantly caused by viral pathogens (4–6). Prior to the emergence of SARS-CoV-2, the most common viral pathogens associated with VRTIs were reported to include rhinovirus, respiratory syncytial virus (RSV), human adenoviruses, parainfluenza virus, influenza virus, and human metapneumovirus (4, 5, 7).

Immunocompromised patients are particularly vulnerable to VRTIs. Patients receiving immunosuppressive therapy or who have disorders associated with immune deficiency, including patients with a history of cancer, solid organ transplant, bone marrow transplant, and primary immunodeficiency disorders may be at a higher risk of VRTIs, complications of infection, and acute or chronic organ transplant rejection (5, 7, 8).

The mainstay therapy for VRTI is supportive care. Patients with severe respiratory infections may present with pneumonia, acute respiratory distress syndrome, decompensated heart failure, or exacerbation of underlying chronic lung disease. These conditions may lead to acute hypoxemia and respiratory failure requiring treatment with non-invasive ventilation, oxygen therapy, and/or invasive ventilation, requiring admission to the intensive care unit (ICU). In addition, fluid management is essential to maintain cardiovascular function. Antiviral therapy is often initiated when available to inhibit viral replication (9).

For immunocompromised patients or other high-risk groups, additional adjuvant therapy with immune-modulating agents, such as corticosteroids, convalescent plasma, or intravenous immune globulin (IVIG), may be considered (9–12). Beneficial clinical outcomes with the use of antiviral agents in combination with intravenous immune globulin have been described in case reports and small prospective trials (13–18). Improvement in respiratory function, oxygenation, and chest radiograph appearance have been reported (14–16), with some trials indicating that antiviral therapy in combination with IVIG may improve survival (17, 18). Although some studies suggest a potential role for use of IVIG in select patients, the impact of IVIG treatment in VRTI is not fully understood. The objective of this study was to assess clinical outcomes as well as hospital resource utilization associated with IVIG use among immunocompromised patients hospitalized for VRTI.

## 2 Methods

### 2.1 Data source and description

Data from the Premier Perspective Hospital dataset January 1, 2011 to December 31, 2017 were used in this analysis. Premier, Inc. is an alliance of more than 4,000 hospitals and health systems. Premier operates an extensive, detailed clinical and financial database that is populated with hospital data received by Premier in connection with the healthcare operations services provided to hospitals through its informatics products (19).

The Premier Healthcare Database contains approximately 25% of annual inpatient admissions data in the United States from more than 1,000 hospitals/healthcare systems. The data, from standard hospital discharge files, contains patient demographics and disease state, and information on billed services, including medications, laboratory, diagnostics, and therapeutic services in de-identified daily patient service records. In addition, information on hospital characteristics, including geographic location, bed size, and teaching status, is also available (19).

All deliverables are de-identified in accordance with HIPAA and therefore the study is exempt from Institutional Review Board review. The time of admission is provided as a month and the time of discharge is provided as a month and year. Day-of-service level details are reported using chronological days (e.g., day 1, day 2) (19).

### 2.2 Study design and measures

For this cross-sectional study design, we used measures occurring within the hospital stay. Hospital data are analyzed at the discharge unit of analysis, not the patient level. A patient may contribute more than one hospitalization to the analysis; however, the measures are limited to the observation within each hospitalization.

The sample included all patient discharges with ICD codes indicating at least one diagnostic code for Acute Viral Respiratory Tract Infection (Appendix A) in addition to one diagnostic code for Any Immune Deficiency (Appendix B). Discharges were then assigned to treatment groups based on the use of IVIG within the first 48 hours of hospital admission.

Independent variables were identified as patient and hospital characteristics that may confound the relationship between IVIG use and hospital-based outcomes among patients admitted for an acute respiratory viral infection. These patient characteristics included demographics as well as documentation of relevant diseases and treatments indicating diseases that may increase bias association between IVIG use and the outcomes. Hospital characteristics included urban versus rural designation, teaching status, and bed size.

All diagnoses were retrieved at the date of discharge while treatments and procedures were identified on the date of administration. Treatments included antivirals and steroids. Procedures identified were mechanical ventilation, indicating respiratory decompensation, as well as transplantation, which is associated with a compromised immune system. Diagnoses included viral infections and chronic respiratory diseases. Treatments and procedures were measured as binary measures of occurrence. Hospital characteristics were measured to address for clustering of more severe patients in larger hospitals as well as for regional differences. Outcomes included measures relevant to hospital resource use and death. Length of stay, both the total hospital as well as ICU stay were measured in days as a count variable whereas death was measured as a binary variable and measured at discharge.

### 2.3 Statistical analysis

All statistical analyses were conducted using SAS version 9.4. All tests were conducted assuming a two-tailed test of significance and alpha level set a-priori at 0.05. Descriptive statistics used Chi-square or Fisher’s Exact test and T-tests where appropriate.

To measure the factors associated with IVIG use across the hospitalization, simple bivariate comparisons across the treatment groups were conducted on measures of central tendencies or proportions using appropriate statistical tests. T-tests were used for the comparison of means between cases and controls and Chi-square or Fischer’s Exact tests were used for the comparison of proportions between cases and controls.

Due to the imbalance of control cases to viral infection cases, an Inverse Probability Weighting (IPW)-based Regression Model was used where 5,000 controls were randomly drawn. The logistic regression model was developed to adjust for factors associated with the probability of IVIG use within 48 hours of admission. A published study with a very similar patient cohort, showed that IVIG use within 48 hours of hospital admission led to significant changes in outcomes (20). The propensity score was then used to weight subsequent models to assess the length of stay (total and ICU) using a negative binomial model and logistical regression for inpatient death.

Due to the observational nature of the data used in this study, IPW was used to effectively mimic randomization of treatment. Initially by predicting the probability of a treatment in a regression model using a propensity score given all known covariates. Then, using the inverse of this propensity score for each observation as a weight within the subsequent regression models for each outcome. This method balances baseline covariates evenly across the study population. This method has been described in greater detail elsewhere (21).

As described in a previous study using similar analyses (20), propensity score weights were generated using logistic regression with IVIG treatment as the outcome with the following covariates: age, gender, transplant status, baseline respiratory support (supplemental oxygen, ventilator use), antiviral medications, viral organism, immunodeficiency type, and a comorbidity index, which is an additive scale including hypertension, diabetes, heart disease, and renal/urinary tract disease.

The effect of IVIG was evaluated with regard to each of the outcomes using a series of IPW multiple regression analyses. Poisson models were used to evaluate hospital length of stay and ICU length of stay. Logistic regression was used for evaluation of death outcomes. In each model, we controlled for age, gender, transplant status, baseline respiratory support, and antiviral medications. A stratified analysis was performed for patients with specific types of transplants and specific types of viral organisms.

## 3 Results

### 3.1 Patient characteristics

**Figure 1:**
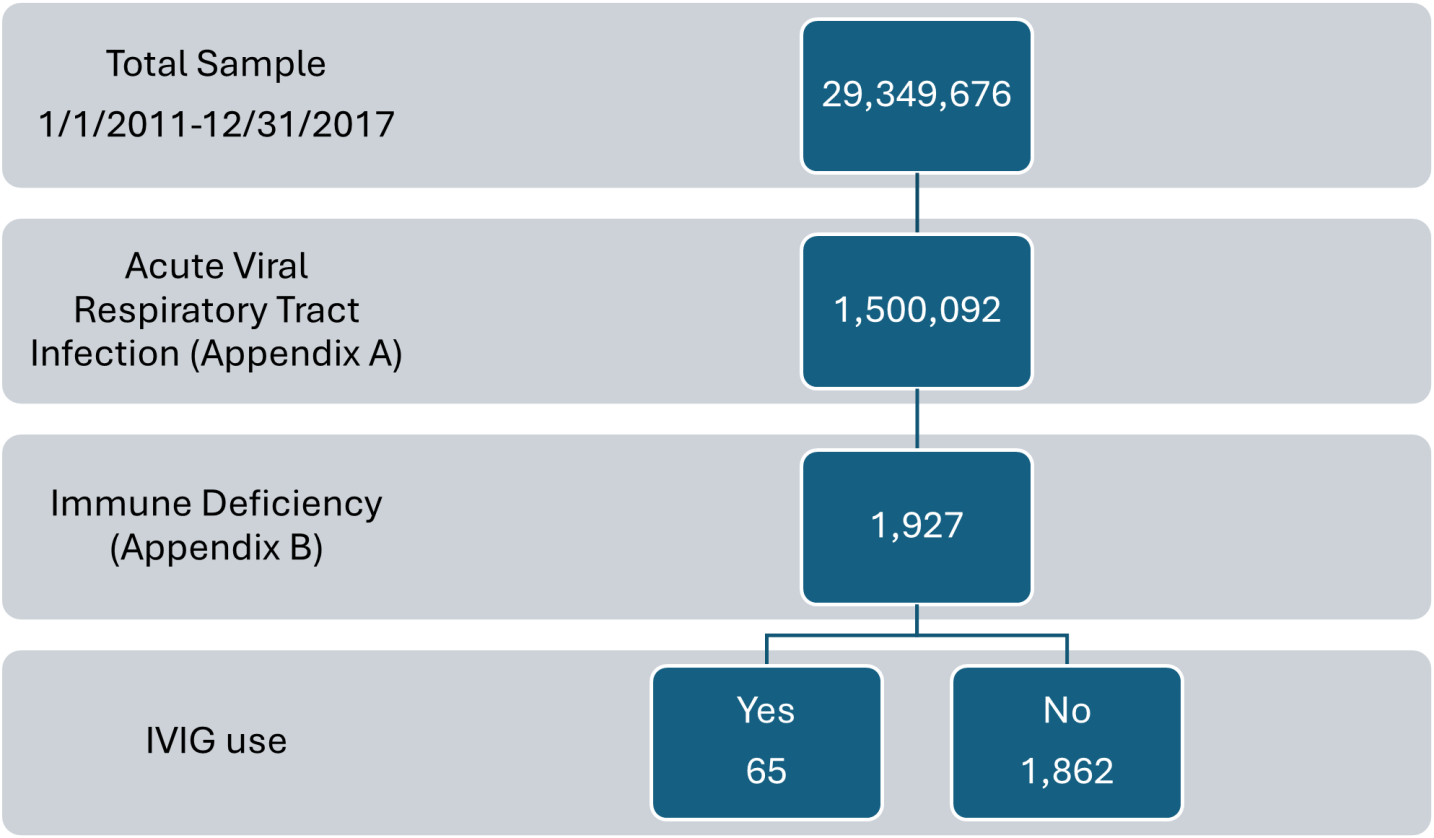
A flow chart of patient disposition selecting patients for acute viral respiratory tract infection, immune deficiency and intravenous immunoglobulin (IVIG).

A total of 1,927 inpatients with immune deficiency and acute respiratory viral infections were analyzed (**Figure 1**). Of those patients, 65 received IVIG during their hospitalization. Patients receiving IVIG were significantly younger in age, more likely to receive ribavirin or other antiviral agents, have a history of primary immune deficiency disorder (PIDD), transplant for bone, lung, or other solid organ transplant, bone marrow transplant, and require intubation during hospitalization compared with patients who did not receive IVIG. Patients who did not receive IVIG were more likely to have cancer or be a recipient of a kidney transplant. (See **Table 1**).

**Table 1:** Patient characteristics of inpatients with any immune deficiency and specific viral respiratory tract infection by IVIG treatment*.

Characteristic	Total Patients		IVIG Group		No IVIG Group		
Total (n, %)	1927	100.00%	65	100.00%	1862	100.00%	p-value
Age (mean, median, SD)	53.88/62/25.61		34.83/36/26.83		54.54/62/26.36		**<0.0001**
Sex							
Male	922	47.85%	36	55.38%	922	49.52%	
Female	940	48.78%	29	44.62%	940	50.48%	
Lung Disease	181	9.39%	2	3.08%	179	9.61%	0.0758
Ribavirin	7	0.36%	4	6.15%	3	0.16%	**<0.0001**
Antiviral	436	22.63%	26	40.00%	410	22.02%	**0.0007**
Prednisone	720	37.36%	26	40.00%	694	37.27%	0.6549
PIDD	266	13.80%	33	50.77%	233	12.51%	**<0.0001**
Transplant – Other	253	13.13%	14	21.54%	239	12.84%	**0.0411**
Transplant – Kidney	134	6.95%	0	0.00%	134	7.20%	**0.0249**
Transplant – Heart	24	1.25%	0	0.00%	24	1.29%	0.357
Transplant – Bone	71	3.68%	6	9.23%	65	3.49%	**0.0157**
Transplant – Lung	20	1.04%	3	4.62%	17	0.91%	**0.0038**
Transplant – Bone Marrow	12	0.62%	5	7.69%	7	0.38%	**<0.0001**
Cancer	1420	73.69%	26	40.00%	1394	74.87%	**<0.0001**
Rhinovirus	567	29.42%	19	29.23%	548	29.43%	0.9723
Metapneumovirus	290	15.05%	12	18.46%	278	14.93%	0.4338
RSV	770	39.96%	24	36.92%	746	40.06%	0.6113
Cystic Fibrosis	0	0.00%	0	0.00%	0	0.00%	NA
Intubation	27	1.40%	4	6.15%	23	1.24%	**0.0009**
Teaching hospital	1086	56.36%	39	60.00%	1047	56.23%	0.5471

*Fischer’s Exact Test or Chi-Square tests for proportions; t-test for means.

IVIG, intravenous immunoglobulin; PIDD, primary immune deficiency disorder; RSV, respiratory syncytial virus; SD, standard deviation.

Values in bold are considered significant (p < 0.05).

Isolated respiratory viruses in the total population were respiratory syncytial virus (39.9%), rhinovirus (29.4%), and metapneumovirus (15.1%). There were no differences in type of virus detected between the two treatment groups (See **Table 1**).

### 3.2 Hospital characteristics

No significant differences in hospital characteristics were found between the two treatment groups. The majority of patients were treated in urban teaching hospitals that had a bed capacity of over 400 beds (See **Table 2**).

**Table 2:** Hospital characteristics of inpatients with acute viral respiratory tract infection by IVIG treatment*.

Characteristic	Total Patients		IVIG Group		No IVIG Group		
Total (n, %)	1927	100.00%	65	100.00%	1862	100.00%	p-value
Urban	1766	91.65%	62	95.38%	1704	91.51%	0.268
Rural	161	8.35%	3	4.62%	158	8.49%	
Teaching Status	1086	56.36%	39	60.00%	1047	56.23%	0.547
Bed Size							0.084
0-99	54	2.8%	0	0.00%	54	2.90%	
100-199	235	12.20%	5	7.69%	230	12.35%	
200-299	222	11.52%	12	18.46%	210	11.28%	
300-399	264	13.70%	4	6.15%	260	13.96%	
400-499	194	10.07%	6	9.23%	188	10.10%	
500+	958	49.71%	38	58.46%	920	49.41%	

*Fischer’s Exact Test for proportions.

IVIG, intravenous immunoglobulin.

### 3.3 Healthcare resource use

Without adjustment, there were no significant differences in total hospital length of stay or inpatient death between the two treatment groups. However, patients who received IVIG had a significantly shorter ICU length of stay (mean ± SD). The mean length of stay in the ICU for patients who received IVIG was 1.63 ± 3.59 days compared with 3.40 ± 7.71 days for those patients who did not receive IVIG (p <0.0001) resulting in a 50% reduction in ICU length of stay. (See **Table 3**).

**Table 3:** Healthcare resource use associated with IVIG treatment among inpatients with any immune deficiency and acute viral respiratory tract infection*.

Characteristic	Total Patients			IVIG Group		No IVIG Group			
Total (n, %)	1927		100.00%	65	100.00%	1862		100.00%	p-value
Length of hospital stay in days (mean/median/SD)	10.90/7/13.46			10.86/7/12.96		12.15/6/23.69			0.662
Length of ICU stay in days (mean/median/SD)	3.34/0/7.62			1.63/0/3.59		3.40/0/7.71			<0.0001
ICU stay	881		45.72%	20	30.77%	861		46.24%	<0.0001
Inpatient death	176		9.13%	4	6.15%	172		8.93%	0.514

*Fischer’s Exact Test for proportions; t-test for means.

ICU, intensive care unit; IVIG, intravenous immunoglobulin; SD, standard deviation.

When adjusting for IVIG use within 48 hours of admission as well as immune system complications, hospital characteristics, relative concomitant medications, and demographics we find that IVIG reduces utilization. The adjustment now revealed a significantly shorter length of hospital stay when patients with VRTI were given IVIG (-3.24; 95% CI, -6.11 to -0.37; p = 0.027). The length of ICU stay was significantly shorter with IVIG use (-1.83; 95% CI, -3.04 to -0.62; p = 0.003). However, no difference was seen with inpatient death rates (See **Table 4**).

**Table 4:** Adjusted outcomes IVIG vs. No IVIG.

Measure	Coefficient	95% CI		p-value
Length of hospital stay in days*	-3.24	-6.11	-0.37	0.027
Length of ICU stay in days*	-1.83	-3.04	-0.62	0.003
Inpatient death^+^	1.08	0.33	3.54	0.903

*Adjustment for immune type (transplant, cancer), RSV, PIDD, age, prednisone, antiviral, ribavirin, urban, teaching, intubation, lung disease using inverse probability weighted regression.

+Adjustment for immune type (transplant, cancer), RSV, PIDD, age, prednisone, antiviral, ribavirin, urban, intubation, lung disease using logistic regression.

CI, confidence interval; ICU, intensive care unit; IVIG, intravenous immunoglobulin; PIDD, primary immune deficiency disorder; RSV, respiratory syncytial virus.

## 4 Discussion

Over 70 years ago, the use of gamma globulin to successfully reduce the frequency of serious bacterial infections was first reported (22). However, there are still limited data on the impact of IVIG use in specific clinical situations. The effects of IVIG administration have been studied as a treatment in RSV, respiratory infections following chemotherapy and hematopoietic transplantation, and the combination of aerosolized ribavirin and IVIG, demonstrating its efficacy in a range of clinical situations (17, 23–25).

The impact of IVIG therapy on hospital length of stay may vary depending on the specific patient population and the condition being treated. Moreover, patient comorbidities and disease severity may play a role in determining outcomes like hospital length of stay. Little is known about IVIG treatment of immunocompromised patients who are hospitalized for VRTI. In this study immunocompromised patients hospitalized with VRTIs, who received IVIG within the first 48 hours of admission, had a shorter length of stay in the ICU and a shorter overall hospital length of stay, which could lead to improved clinical outcomes and significant cost savings. A shorter length of stay following IVIG treatment has also been reported in COVID-19 patients in a prospective randomized trial and in a meta-analysis of critically ill COVID-19 patients (26, 27). Conversely, in other meta-analyses of COVID-19 patients, IVIG demonstrated no benefit with regard to patient outcomes and hospital length of stay or even an increase in hospital length of stay (28, 29). The inconsistency in the literature with regard to the efficacy of IVIG treatment in influencing length of stay in COVID-19 patients may be related to how recent COVID-19 became a major public health problem and more research is needed to improve clarity.

Patients with an RSV infection made up almost 40% of our sample. Previous studies on the efficacy of IVIG treatment in RSV patients focused on pediatric populations and although outcomes were improved, hospital length of stay was not shorter a result supported in a recent Cochrane Systematic review (23, 30, 31). In the present study, patients treated with IVIG were adults and therefore the combination of these studies suggests that IVIG treatment can be efficacious in a broad age range, but this may vary depending on the specifics of patient populations. Reduced hospital length of stay with IVIG treatment has also been reported in relation to other conditions. For example, when patients with Guillain-Barré Syndrome were treated with IVIG, mechanical ventilation weaning was improved, and hospital length of stay was significantly shorter compared to patients treated with plasma exchange (32, 33). This suggests that IVIG treatment may be beneficial in a range of infection types.

In this study, patients who were treated with IVIG were more likely to have had a transplant of some kind. Use of IVIG desensitization in renal transplant recipients was associated with a significantly improved survival (34, 35). Moreover, IVIG treatment in liver transplant patients may also reduce organ rejection, which is important considering the limited availability of viable organs (36, 37). Improved transplant patient survival and reduced organ rejection rates could be considered a proxy for the amount of time a patient spends in the hospital and therefore we provide further evidence to support this notion.

One interesting outcome of this study was that IVIG treatment reduced hospital length of stay without improving mortality rates, which does not align with previous analyses. However, while speculative, this result may be biased since patients with more severe illness were more likely to be treated with IVIG. Conversely, in a recent meta-analysis, mortality in streptococcal toxic shock syndrome patients treated with clindamycin and IVIG (vs. clindamycin only) reduced by more than half (38). Further, in severely ill COVID-19 patients, high dose IVIG reduced mortality rates (39). Overall, evidence suggests that IVIG treatment may be associated with a lower risk of mortality in some clinical settings. However, the effect of IVIG may depend on the underlying condition, the indication for treatment, and the individual patient as suggested by the differences in the result of this study compared to previous works.

A primary focus of this study was the use of IVIG in patients with VRTIs, for which data is lacking. Interest in the use of IVIG in the treatment of VRTI is based on the recognition of the immunomodulatory effects of high-dose IVIG therapy. The proposed mechanisms of immunomodulation are mediated by the two functional domains of IgG: the F(ab)’2 fragment that is responsible for specific antigen binding and the Fc fragment that is responsible for Fc receptor and complement binding. F(ab)’2-mediated mechanisms of immunomodulation include passive immunity through the neutralization of pathogenic antigens and an anti-inflammatory effect through neutralization of pro-inflammatory cytokines and chemokines. Fc-mediated anti-inflammatory effects are thought to result from the saturation of Fc receptors (FcγRs) and upregulation of the inhibitory Fc receptor (FcγRIIB) (40). Viral respiratory tract infections have been associated with excessive production of pro-inflammatory cytokines, which can mediate damage to normal tissue in the lungs and other organs. In these hyper-immune conditions, high-dose IVIG may play an important role in immune modulation (40).

Based on the proposed mechanisms of action, high-dose IVIG has been used clinically in the treatment of VRTI. Although the most effective timing of therapy is unclear, some studies suggest that early initiation of immune modulators (corticosteroids or IVIG) in addition to antivirals may provide clinical benefit (41, 42).

While there is no definitive evidence from randomized clinical trials to support the use of IVIG in the treatment of VRTI, several open-label studies describe the potential benefits of high-dose IVIG therapy in combination with antiviral agents in immunocompromised patients who are at high risk for VRTI. Studies suggest that early treatment of upper respiratory RSV infection with aerosolized ribavirin and IVIG may reduce the frequency of progression to pneumonia and death in adult transplant recipients (17, 18). Similarly, studies examining outcomes in bone marrow transplant patients with respiratory CMV infection found that treatment with ganciclovir and IVIG was associated with clinical improvement and survival (43, 44).

The examination of healthcare resource utilization, as described in this study, provides additional data to evaluate the impact of IVIG therapy in the setting of VRTI. The reduction in ICU length of stay and overall hospital length of stay suggests beneficial resource utilization outcomes with IVIG therapy that may indirectly reflect positive clinical outcomes for immunocompromised patients with VRTI.

There are potential limitations in this study. Hospital chargemaster data have the potential for errors and miscoding, which could introduce misclassification bias into the study by incorrectly identifying patients with IVIG treatment. Patients may be duplicated as the data are cross-sectional and each occurrence represents a discharge, not a patient. Therefore, patients having multiple hospitalizations may be occurring in the study analysis as separate discharges. And finally, the dosage of IVIG is not available in the dataset, so it is unknown whether a patient received high-dose or low-dose IVIG and how that might have affected the outcomes.

In summary, immunocompromised patients with VRTI who receive IVIG within 48 hours of ICU admission may have a shorter ICU length of stay and shorter overall hospital length of stay. These findings suggest possible beneficial health outcomes for the patient as well as for hospital resource utilization. Further research via prospective clinical trials is needed to explore the potential impact of IVIG in this setting on other health outcomes and resource use to identify specific patients who may benefit from IVIG therapy as part of their treatment for viral respiratory tract infection.

## Data Availability

The data supporting the conclusions of this article is available from the Premier Healthcare Database. Information on the Premier database is available at: PremierHealthcareDatabaseWhitepaper.pdf (premierinc.com).

## APPENDIX A

**Table:** Acute Viral Respiratory Tract Infections ICD Codes.

Viral Respiratory Tract Infections	ICD-9	ICD-10
RSV (acute bronchiolitis due to respiratory syncytial virus)	466.11	J21.0
RSV (respiratory syncytial virus infection)		B97.4
RSV (respiratory syncytial virus pneumonia)	480.1	J12.1
RSV bronchitis		J20.5
Positive sputum culture for RSV		R84.5
Acute bronchitis due to parainfluenza virus		J20.4
Acute bronchitis due to Rhinovirus		J20.6
Parainfluenza virus bronchopneumonia		J12.2
Parainfluenza virus infection		B33.8
Parainfluenza type 1 infection; Rhinovirus		B34.8
Acute bronchiolitis due to human metapneumovirus		J21.1
Pneumonia due to human metapneumovirus		J12.3
Infection due to human metapneumovirus		B97.81
Pneumonia due to adenovirus	480.0	J12.0

ICD, International Classification of Diseases; RSV, respiratory syncytial virus.

## APPENDIX B

**Table:** **Immune Deficiency ICD Codes**.

Immune Deficiency Diagnosis	Sample	ICD 9	ICD 10
Hospitalized for chemotherapy treatment	1	V58.11	Z51.11
Complication of chemotherapy	1		T88.7XXA
Cancer	1	199.1	C80.1
History of immunosuppressive therapy	1	V87.46	Z92.25
Transplant, organ	1	V42	Z94.9
Kidney replaced by transplant	1	V42.0	Z94.0
Heart replaced by transplant	1	V42.1	Z94.1
Bone replaced by transplant	1	V42.4	Z94.81
Lung replaced by transplant	1	V42.6	Z94.2
Liver replaced by transplant	1	V42.7	Z94.4
Other specified organ or tissue replaced by transplant	1	V42.8	
Unspecified organ or tissue replaced by transplant	1	V42.9	
Bone marrow replaced by transplant	1	V42.81	
Transplant follow up	1		Z48.298
Transplant recipient	1		Z94.89
History of bone marrow transplant	1		Z94.81
History of organ or tissue transplant	1		Z94.9
Complications, organ transplant	1	996.80	T86.90
Primary Immunodeficiency Disorder	1, 2	279.0X, 279.12, 279.2X	D80.1, D80.2, D80.3, D80.4, D80.5, D80.7, D81.0, D81.1, D81.2, D81.6, D81.7, D81.89, D81.9, D82.0, D83.0, D83.1, D83.2, D83.8, D83.9
Immunodeficiency disorder due to antibody deficiency	1		D80.6
Immunodeficiency due to chemotherapy	1	V58.11	Z79.899
Immunodeficiency secondary to neoplasm	1		D84.8, D49.9
CVID (Common Variable Immunodeficiency)	1, 2	279.06	D83.9
Deficiency, humoral	1, 2	279.00	
Immunodeficiency associated with major defect	1		D82.9
Immunodeficiency with predominantly antibody defects	1		D80.9
Combined Immunodeficiency, unspecified	1, 2	279.2	D81.9
Disorder of immune system	1		D89.9
Disorder of Lymphatic system	1		I89.9
Encounter for antineoplastic immunotherapy	1	V58.12	Z51.12

ICD, International Classification of Diseases.
